# CMV-specific T cell isolation from G-CSF mobilized peripheral blood: depletion of myeloid progenitors eliminates non-specific binding of MHC-multimers

**DOI:** 10.1186/s12967-014-0317-8

**Published:** 2014-11-19

**Authors:** Lorea Beloki, Miriam Ciaurriz, Cristina Mansilla, Amaya Zabalza, Estela Perez-Valderrama, Edward R Samuel, Mark W Lowdell, Natalia Ramirez, Eduardo Olavarria

**Affiliations:** Oncohematology Research Group, Navarrabiomed - Miguel Servet Foundation, Irunlarrea 3, 31008 Pamplona, Spain; Department of Haematology, University College London Medical School, University College London, London, UK; Department of Haematology, Complejo Hospitalario de Navarra, Navarra Health Service, Pamplona, Spain

**Keywords:** Allogeneic hematopoietic stem cell transplantation, Cytomegalovirus-specific cytotoxic T cells, MHC-multimers, Granulocyte-colony stimulating factor, Immunotherapy

## Abstract

**Background:**

Cytomegalovirus (CMV)-specific T cell infusion to immunocompromised patients following allogeneic Hematopoietic Stem Cell Transplantation (allo-HSCT) is able to induce a successful anti-viral response. These cells have classically been manufactured from steady-state apheresis samples collected from the donor in an additional harvest prior to G-CSF mobilization, treatment that induces hematopoietic stem cell (HSC) mobilization to the periphery. However, two closely-timed cellular collections are not usually available in the unrelated donor setting, which limits the accessibility of anti-viral cells for adoptive immunotherapy. CMV-specific cytotoxic T cell (CTL) manufacture from the same G-CSF mobilized donor stem cell harvest offers great regulatory advantages, but the isolation using MHC-multimers is hampered by the high non-specific binding to myeloid progenitors, which reduces the purity of the cellular product.

**Methods:**

In the present study we describe an easy and fast method based on plastic adherence to remove myeloid cell subsets from 11 G-CSF mobilized donor samples. CMV-specific CTLs were isolated from the non-adherent fraction using pentamers and purity and yield of the process were compared to products obtained from unmanipulated samples.

**Results:**

After the elimination of unwanted cell subtypes, non-specific binding of pentamers was notably reduced. Accordingly, following the isolation process the purity of the obtained cellular product was significantly improved.

**Conclusions:**

G-CSF mobilized leukapheresis samples can successfully be used to isolate antigen-specific T cells with MHC-multimers to be adoptively transferred following allo-HSCT, widening the accessibility of this therapy in the unrelated donor setting. The combination of the clinically translatable plastic adherence process to the antigen-specific cell isolation using MHC-multimers improves the quality of the therapeutic cellular product, thereby reducing the clinical negative effects associated with undesired alloreactive cell infusion.

## Background

Allogeneic hematopoietic stem cell transplantation (allo-HSCT) allows the recovery of a sick hematopoietic system affected by congenital or acquired severe disorders [1]. Peripheral blood stem cells (PBSCs) are the main hematopoietic stem cell source [2], recombinant granulocyte-colony stimulating factor (G-CSF; Filgrastim) being clinically used for the mobilization of hematopoietic stem cells (HSCs) to the periphery. This treatment enriches the sample in neutrophils, monocytes, lymphoid and myeloid progenitor cells at different stages of maturation that will be differentiated into monocytic and granulocytic lineages [3-5].

Studies of immune reconstitution after allo-HSCT have identified a decisive role of CD8+ cytotoxic T lymphocyte (CTL) recovery in preventing the development of viral diseases [6]. Amongst them, CMV infection remains a major complication in recipients following allo-HSCT, and the adoptive transfer of CMV-specific T cells has shown successful clinical results [7-12]. Several strategies have been used for anti-CMV T cell manufacture [13-17]. MHC-multimers allow the direct selection of antigen-specific CD8+ T cells with no need for long-term *in vitro* culture, offering a direct and fast selection strategy [18]. This avoids the functional damaging effects of the expansion, thereby preserving the survival potential and cellular properties of the therapeutic product [19-21].

Historically, the manufacture of virus-specific T cells for adoptive immunotherapy has involved the use of donor lymphocytes collected from a steady-state leukapheresis obtained from an additional apheresis prior to the G-CSF administration for HSC mobilization. G-CSF has previously been shown to induce immunologic tolerance; it promotes T helper type 2 (Th2) and regulatory T cell differentiation and downregulates genes associated with Th1 cells, cytotoxicity, antigen presentation and graft versus host disease (GvHD) [22-25]. In spite of the above described immunosuppressive effects of G-CSF treatment, recently some authors have successfully generated competent CMV-specific T cells from G-CSF mobilized apheresis samples [26,27]. CMV-specific T cell manufacture from the same G-CSF mobilized collection used to obtain HSCs would abrogate the need for successive donations, assuring the availability of an anti-viral cell product in the unrelated donor setting while minimizing costs and discomfort for the donor.

Therefore, we aimed to improving CMV-specific T cell isolation from G-CSF mobilized donors using MHC-multimers. In the present study, we have developed a method to avoid non-specific binding of multimers to potentially damaging cell subsets by using a physical procedure based on plastic adherence [28]. In this way, we have managed to minimize the non-specific binding of multimers and eventually obtain a more pure cellular product safer for infusion.

## Methods

### Donor population and ethical statement

This study was approved by the Institutional Review Board at Complejo Hospitalario de Navarra (CHN), and all donors gave informed consent prior to enrolment.

11 subjects who were stem cell donors at CHN for allo-HSCT were recruited. All were CMV-seropositive and carried the HLA-A*02:01 allele. HLA-I typing was done in the Immunology Unit and the serological analysis for CMV was obtained from the Microbiology Service of the CHN.

### PBSC mobilization and collection

Cells were collected from donors who received 10 μg/kg/day of recombinant G-CSF (Filgrastim, Sandoz Biopharmaceuticals, Paris, France) every 12 hours starting five days before collection. Leukapheresis were performed with a COBE Spectra continuous flow blood cell separator (COBE Spectra apheresis system, Caridian BCT, Lakewood, CO, USA). Cell products, anticoagulated with ACD-A, were collected with a 1.1 ml/min flux in a 500 ml container, from which an aliquot of 0.5 ml was used to perform the experiments. Peripheral blood mononuclear cells (PBMCs) were isolated by Ficoll-Paque density gradient centrifugation (GE Healthcare Bio-Sciences, Uppsala, Sweden) and counted in Neubauer hemocytometer using 0.4% trypan blue staining (Gibco, Carlsbad, CA).

### Enrichment of lymphocyte populations by plastic adherence

2.25 × 10^7^ cells were suspended in 45 ml of X-VIVO 15 Serum-free cell medium w/o supplements (Lonza, Basel, Switzerland) in a sterile 225 cm^2^ A/N flask with CellBIND Surface (Corning, Corning, NY) for 1 hour at 37°C and 5% CO_2_. Non-adherent cells were carefully collected by aspiration to avoid the disruption of the adherent cellular populations. Obtained cells were washed with Dulbecco’s phosphate buffered saline (dPBS, Sigma-Aldrich, St. Louis, MO) before quantification and cytometric analysis.

### Phenotypic characterization of unmanipulated PBMCs and non-adherent cell product

Fresh G-CSF mobilized PBMCs and the cellular harvest obtained after adherent cell removal were phenotypically characterized.

#### Characterization of leukocyte subpopulations

1 × 10^6^ cells were stained with anti-human CD3-V450 (BD Biosciences, San Jose, USA), CD8-FITC (BioLegend, San Diego, USA), CD14-PE (BioLegend), CD45-PerCP-Cy5.5 (BioLegend), and CD4-APC (BioLegend). Incubation was carried out for 15 minutes in the dark, cells were washed once and resuspended in dPBS prior to cytometer acquisition.

#### CMV-specific CD8+ T cell quantification

5 μl of PE-labelled Pentamer (PM; HLA-A*0201/CMV Pentamer, Proimmune, Oxford, United Kingdom) was added to 1 × 10^6^ cells in a final volume of 50 μl. After an incubation of 10 minutes in the dark, cells were stained with CD3-V450, CD8-FITC, 7-AAD (BD), and CD45-APC-H7 (BD). Samples were incubated with monoclonal antibodies for 15 minutes in the dark, washed once and resuspended in dPBS prior to cytometer acquisition.

### CMV-specific T cell selection

From 5 G-CSF mobilized PBMCs before and after the adherent cell removal, 1 × 10^7^ cells were stained with 50 μl PE-labelled PM during 20 minutes at 4°C in the dark. After a wash, 20 μl of anti-PE microbeads (Miltenyi Biotec, Bergisch Gladbach, Germany) were added to a final volume of 80 μl, followed by 20 minute incubation at 4°C in the dark. Afterwards, PM+ cells were isolated using a Possel_ds selection program on an AutoMACS Pro separator (Miltenyi Biotec).

### Acquisition and analysis by Flow Cytometry

Samples were acquired in a FACSCanto II equipment (BD), FACSDiva 6.0 software (BD) was used for acquisition process and FlowJo version 10 (TreeStar Inc., Ashland, OR, USA) for cellular analysis. For the quantification of leukocyte subpopulations 50,000 total events were acquired, and CD3+CD8+, CD3+CD4+, and CD14+ frequencies were defined. For the CMV-specific T cell quantification 500,000 total events were acquired, and frequencies of total AAD+ and PM+CD3+CD8+ were determined. Only cells clustering with forward and side scatter properties of leukocyte subpopulations were included in the analysis, and the percentages were given from the CD45+ cell gate.

### Statistical analysis

Data are represented as median (IQR). Wilcoxon signed-rank test was used for paired comparisons and the significance level was fixed to p < 0.05. Statistical analysis was done using SPSS17 software package.

## Results

### CD8+ cell enrichment and unwanted cell depletion after adherence

47.8% (36.0 – 55.0) of all PBMCs present in the mobilized apheresis expressed the CD14 marker. After the adherence process, CD14+ cells were significantly reduced to 2.1% (1.3 – 6.1) (p = 0.005).

**Figure 1:**
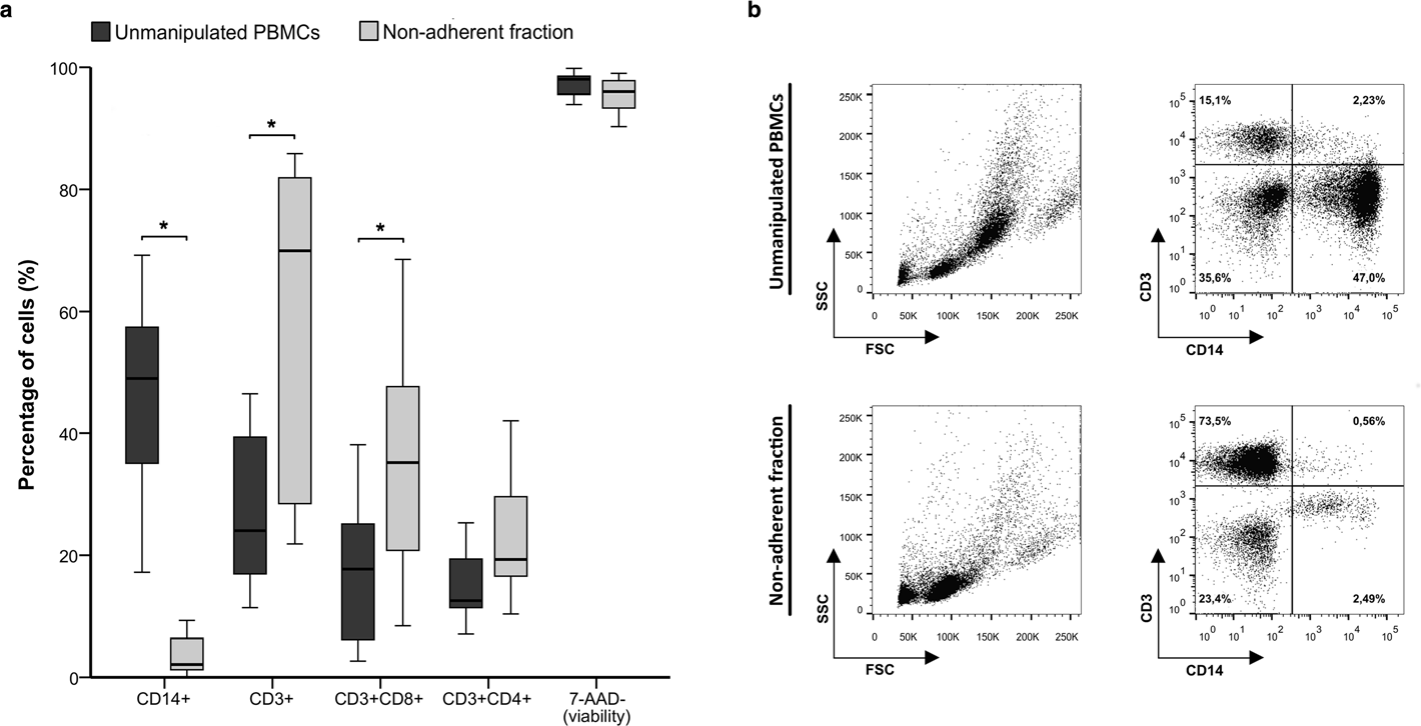
**Phenotypic analysis of cells before and after the adherence process.** Plastic adherence method was applied to PBMCs from G-CSF mobilized donors (*n = 11*) and CD14, CD3, CD8, CD4 and 7-ADD expression of the unmanipulated PBMCs and the non-adherent cells were analyzed by flow cytometry **(a)**. Representative figure of the products before and after the adherent process **(b)**. Cells are presented from the CD45+ cell gate.

In the original sample, 24.0% (18.0 – 37.1) expressed CD3+, from which 17.7% (6.8 – 25.1) were CD8+ and 12.5% (11.4 – 19.4) were CD4+ cells. In the non-adherent cellular product CD3+ cells were significantly increased to 69.9% (31.9 – 79.2) (p = 0.007) and CD8+ subpopulation accordingly rose to 35.2% (23.8 – 45.2) (p = 0.009). CD4+ cell percentage remained at 19.3% (16.6 – 29.6), without reaching statistical significance (p > 0.05) (Figure 1a,b, Table 1).

**Table 1:** **Phenotypic characterization and CMV-specific CTL isolation of unmanipulated PBMCs and the non-adherent fraction**

	**Unmanipulated PBMCs**	**Non-adherent fraction**	**p**
	**Leukocyte subpopulations**		
**CD14+ cells**	47.8% (36.0 – 55.0)	2.1% (1.3 – 6.1)	0.005
**T lymphocytes** (CD3+)	24.0% (18.0 – 37.1)	69.9% (31.9 – 79.2)	0.007
**CTLs** (CD3 + CD8+)	17.7% (6.8 – 25.1)	35.2% (23.8 – 45.2)	0.009
**Helper T cells** (CD3 + CD4+)	12.5% (11.4 – 19.4)	19.3% (16.6 – 29.6)	0.208
**Viability** (7-AAD-)	98.0% (95.6 – 98.5)	96.0% (93.4 – 97.8)	0.066
	**PM staining: specific and non-specific binding**		
**CMV-specific CTLs** (CD3 + CD8 + PM+)	0.14% (0.06 – 0.62)	0.65% (0.24 – 1.51)	0.003
**Non-specific PM binding** (CD8-PM+)	0.56% (0.41 – 0.86)	0.16% (0.12 – 0.37)	0.003
	**CMV-specific CTL isolation**		
**Purity**	20.8% (6.9 – 61.7)	76.0% (32.7 – 83.7)	0.043
**Yield**	38.6% (32.9 – 44.8)	42.1% (23.4 – 84.1)	0.893

Cell subsets, CMV-specific CTLs and non-specific PM staining were analyzed in the unmanipulated PBMC sample and in the non-adherent cells (*n = 11*). CMV-specific CTLs were isolated from unmanipulated PBMCs and non-adherent fraction, and purity and yield of the obtained cellular product were determined (*n = 5*). Comparison was done with the Wilcoxon signed-rank test and significance level was fixed to p < 0.05.

Percentages of different cell population recovery rates were calculated comparing the absolute number before adherence and the cells recovered in the non-adherent cellular fraction. 98.5% (96.3 – 99.6) of CD14+ cells from the unmanipulated PBMC sample were lost after the adherence process. In comparison, 63.7% (33.9 – 70.0) of the T lymphocyte population were collected in the non-adherent fraction, while 65.1% (31.9 – 81.3) and 53.2% (29.2 – 62.6) of the CD8+ and CD4+ cells were recovered, respectively.

7-AAD dye was used to assess cell viability before and after adherence. In the original mobilized sample 98.0% (95.6 – 98.5) of cells were viable, whereas after adherence the viability was similar, 96.0% (93.4 – 97.8) (p > 0.05).

### CMV-specific T cell enrichment and non-specific binding loss by plastic adherence

**Figure 2:**
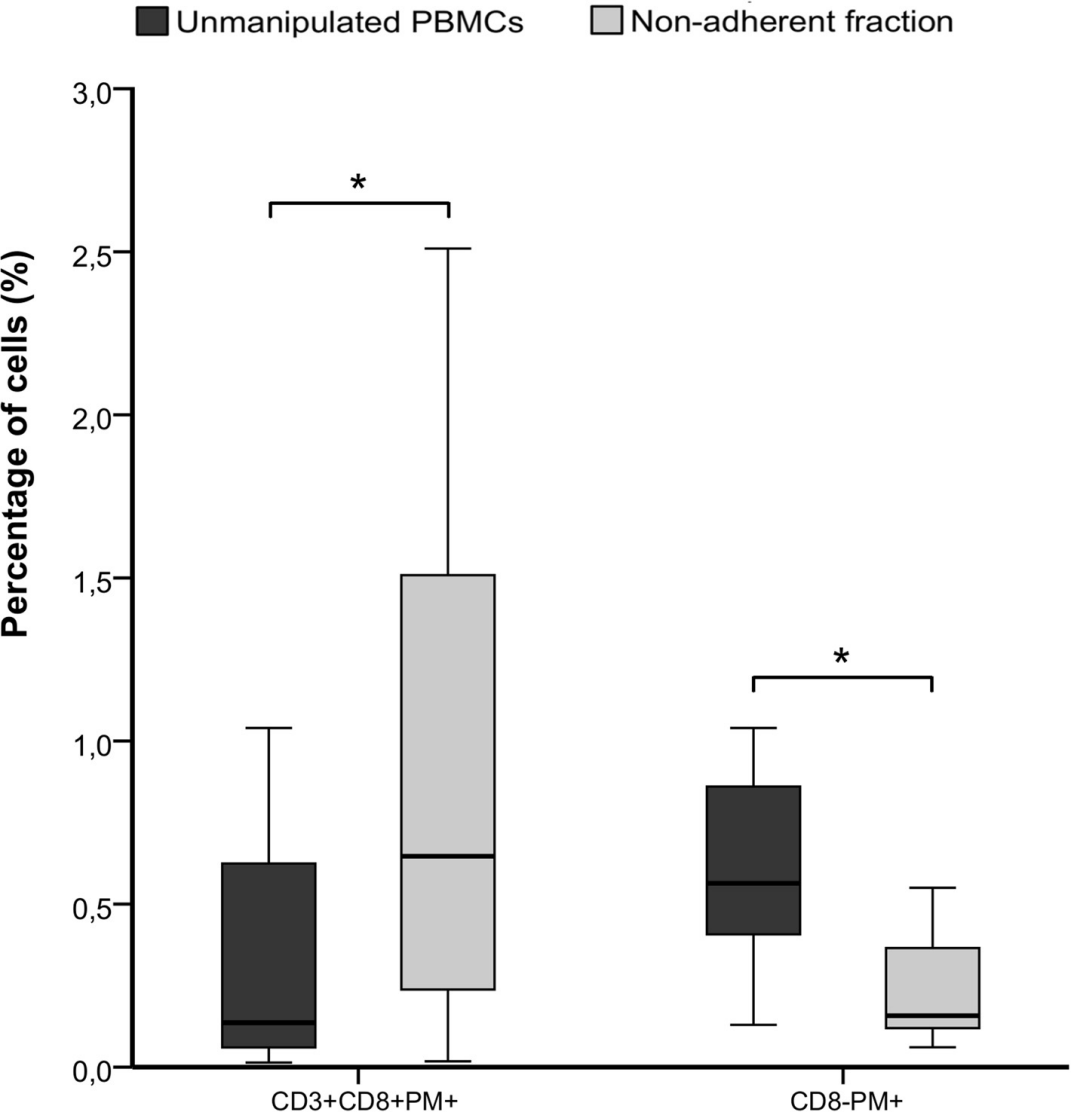
**PM staining before and after the adherence process.** Plastic adherence method was applied to PBMCs from G-CSF mobilized donors (*n = 11*) and PM+ cells were quantified in unmanipulated PBMCs and the non-adherent cell fraction. Specific (CD3+CD8+PM+) and non-specific (CD8-PM+) PM binding was analyzed. Percentages were analyzed from CD45+ cell gate.

Prior to adherence 0.14% (0.06 – 0.62) of PBMCs were specific for CMV (CD3+CD8+PM+), whereas after the adherence process this subpopulation was enriched to 0.65% (0.24 – 1.51) (p = 0.003) in the non-adherent fraction (Figure 2, Table 1). The recovery rate of CMV-specific CTLs in the adherence process was 84.6% (56.3 – 88.7).

In the same way, the non-specific binding of multimers to unwanted CD8- cells was diminished. In the original apheresis sample 0.56% (0.41 – 0.86) CD8-PM+ were detected, while after adherence the non-specific CD8-PM+ cells were reduced to 0.16% (0.12 – 0.37) (p = 0.003).

### Optimization of CMV-specific T cell isolation by magnetic selection

**Figure 3:**
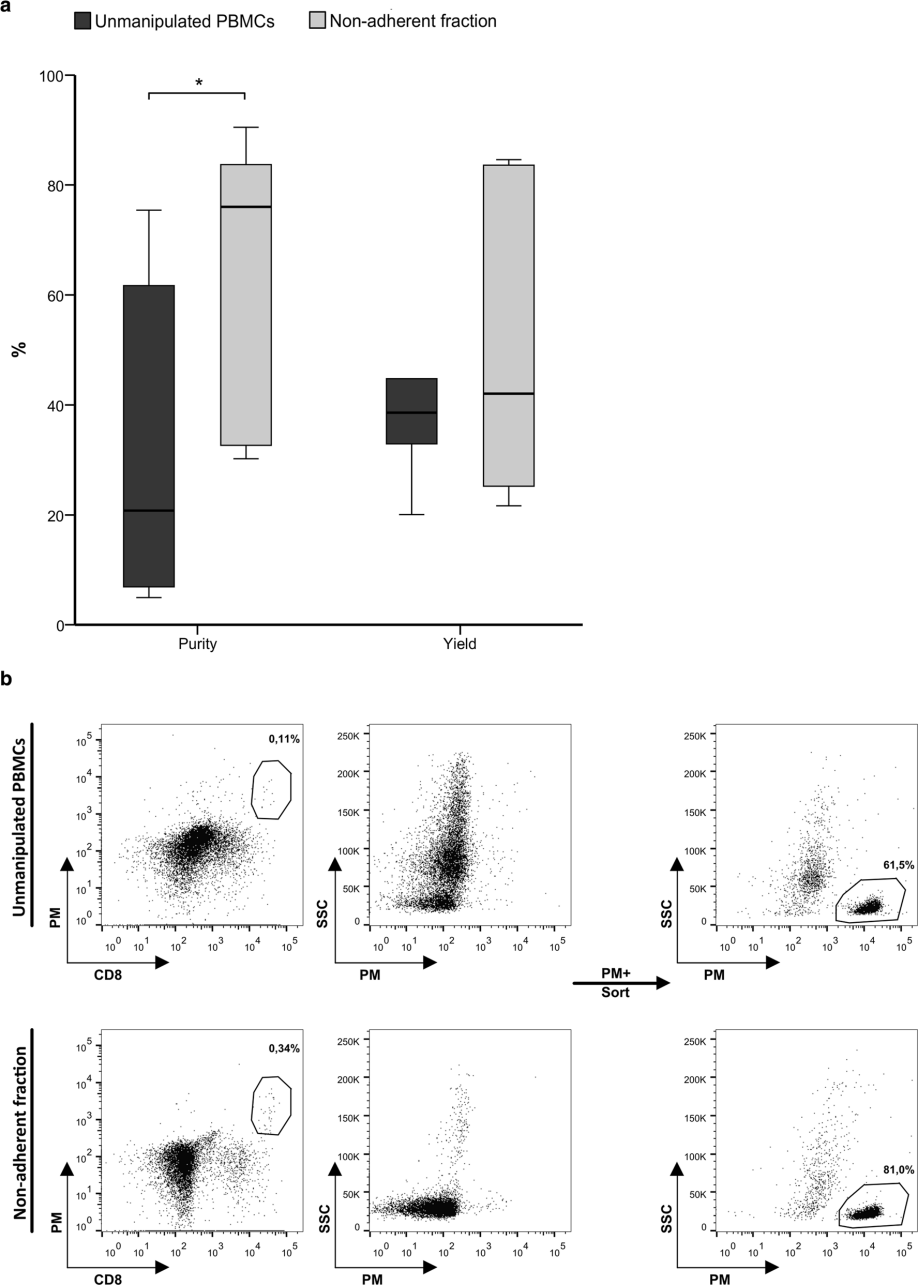
**CMV-specific CTL isolation using Pentamer.** CMV-specific CTLs were isolated from unmanipulated PBMCs and the non-adherent fraction using PM (*n = 5*) and the purity and yield of the process were determined **(a)**. Representative histograms of CMV-specific CTL isolation **(b)**. Displayed cells were previously gated on CD45+ cells.

The purity of the obtained sample was determined as the percentage of PM+ cells in the product, and the yield was defined as the absolute number of PM+ cells present in the positive fraction as a proportion of the absolute number of PM+ cells in the sample before isolation. Using unmanipulated PBMCs, the median purity of the cellular product was 20.8% (6.9 – 61.7) and the yield was 38.6% (32.9 – 44.8). In comparison, the purity of the positive fraction of the isolation using the non-adherent fraction was significantly increased to 76.0% (32.7 – 83.7) (p = 0.043) while the yield was 42.1% (23.4 – 84.1) (p > 0.05) (Figure 3a,b, Table 1).

## Discussion

Alloreactive donor effector cells have been identified as key players in the GvH reaction [29,30]. In this sense, donor derived monocytes have been implicated in the pathophysiology of clinical GvHD [31,32]. Therefore, it is important to assure that the product to be adoptively transferred contains a pure virus-specific T cell population with high specificity.

Since the generation of anti-viral cell products from G-CSF mobilized apheresis samples offers great logistical advantages especially in the unrelated donor setting, we assessed the direct isolation of CMV-specific CTL from G-CSF mobilized PBMCs using MHC-multimers. Donors that participated in this study were mobilized with a biosimilar of G-CSF, with similar effects in comparison to original G-CSF [33]. However, in our first approaches we found a high proportion of MHC-multimer binding to CD8- cells and a low purity in the isolated cell product. In this sense, recent studies have shown that background levels of multimer staining are higher in G-CSF mobilized samples compared to non-mobilized ones [22]. Multimers can join non-specifically to Fc receptors (FcRs) [34], that are mainly expressed on monocytes, dendritic cells, neutrophils, and eosinophils [35]. The up-regulation of FcRI and FcRIII in neutrophils and monocytes induced by G-CSF treatment [36,37] could explain the high background levels described in MHC-multimer staining when G-CSF mobilized samples are used. Furthermore, multimers have been described to bind non-specifically to CD14+ cells [34,38], and G-CSF treatment in healthy individuals results in an increased expression of the CD14 antigen on neutrophils while maintaining its expression on monocytes [39,40]. Therefore, we found the necessity to develop an approach that could avoid the non-specific binding of multimers and the subsequent isolation of potentially alloreactive cells.

A simple process based on plastic adherence reduced all unwanted cellular subsets from mobilized samples due to the fact that hematopoietic progenitors [41], monocytes [42], and neutrophils [39,43], enriched in peripheral blood in response to G-CSF treatment, have the ability to adhere to plastic surfaces. At the same time the proportion of CTLs, which do not adhere to the plastic, was significantly increased in the non-adherent product. Accordingly, after the adherence process the sample was enriched in CMV-specific CTLs and the binding of the multimers to CD8- cells was reduced, which consequently provoked an increase in the efficacy of the subsequent CMV-specific T cell magnetic selection process.

Clinical protocols that infused CMV-specific CTLs obtained through MHC-multimer isolation to avoid CMV reactivation after allo-HSCT have described that an infusion of less than 1 × 10^4^ cells/kg resulted in a considerable expansion of CMV-specific CTLs *in vivo* and was able to control CMV viremia [11,12]. According to our results, these cell numbers could be manufactured from an aliquot of the original G-CSF mobilized PBSC graft (approximately 5 ml, depending on the cellularity and CMV-specific T cell percentage in the G-CSF mobilized harvest). However, for the routine applicability of the method described in this study, the adherence process would be more easily performed using Hyperflask devices (Corning), with a median of 8 flasks (range 2–13) necessary to obtain the required CMV-specific CTL numbers. At the same time, after the adherence process the multimer quantity required to isolate the same amount of specific cells would be reduced, with the associated decrease in the cost of the procedure.

## Conclusions

In the present study we have addressed the high non-specificity present in the CMV-specific T cell isolation using MHC-multimers from G-CSF mobilized donors using an easy, safe and cheap physical procedure, which is readily translatable into the clinic by using Hyperflask devices and GMP grade MHC-multimers. The implementation of this simple method to use G-CSF mobilized PBMCs as starting material for the manufacture of anti-viral cells, apart from reducing costs, would facilitate the accessibility of antigen-specific cellular products for adoptive immunotherapy following allo-HSCT, widening the number of patients that could benefit from this successful therapy.

## Abbreviations

Allo-HSCT: Allogeneic hematopoietic stem cell transplantation
CTL: Cytotoxic T cells
FcR: Fc receptor
G-CSF: Granulocyte-colony stimulating factor
GvHD: Graft versus host disease
HSC: Hematopoietic stem cell
PBSC: Peripheral blood stem cell
Th: Helper T cell
